# Exosome Mimetics-Loaded Hydrogel Accelerates Wound Repair by Transferring Functional Mitochondrial Proteins

**DOI:** 10.3389/fbioe.2022.866505

**Published:** 2022-05-20

**Authors:** Jie Zhu, Zhixiao Liu, Ling Wang, Qishu Jin, Yunpeng Zhao, Antong Du, Neng Ding, Yue Wang, Hua Jiang, Lie Zhu

**Affiliations:** ^1^ Department of Plastic and Reconstructive Surgery, Second Affiliated Hospital of Naval Medical University, Shanghai, China; ^2^ Department of Histology and Embryology, College of Basic Medicine, Naval Medical University, Shanghai, China; ^3^ Stem Cell and Regeneration Medicine Institute, Research Center of Translational Medicine, Naval Medical University, Shanghai, China; ^4^ Department of Histology and Embryology, Harbin Medical University, Harbin, China; ^5^ Shanghai Key Laboratory of Cell Engineering, Shanghai, China; ^6^ Department of Plastic and Reconstructive Surgery, Shanghai East Hospital, Tongji University, Shanghai, China

**Keywords:** exosome mimetics, hydrogel, wound healing, oxidative phoshorylation, exosome

## Abstract

Loading human umbilical mesenchymal stem cell (hUMSC) derived exosomes onto hydrogel scaffolds is a strategy for rapid wound healing. The clinical application of exosomes is hindered by low production, and exosome mimetics could be substituted for exosomes. Here, the therapeutic effects of exosome-loaded hydrogels and exosome mimetic-loaded hydrogels on wounds are evaluated. Our results revealed that exosome mimetic-loaded hydrogels promote wound healing more efficiently than exosome-loaded hydrogels. Exosome mimetics can promote the proliferation and migration of dermal fibroblasts (hDF-a) cells *in vitro*. To investigate how exosome mimetics play a role, proteomics analysis was applied, and the obtained results suggested that exosome mimetics significantly enrich mitochondrial-derived oxidative phosphorylation-related proteins in comparison to exosomes. Overall, our work envisages the emerging potential of exosome mimetics, which take the advantage of exosomes and can be promising candidates for exosomes. It also suggests that hUMSC-derived exosome mimetic-loaded hydrogels have remarkable prospects for clinical application.

## Introduction

Skin wound healing, which is essential for the survival of organisms, is a natural process that restores the integrity of normal skin and relieves injury (Han and Ceilley, 2017). However, inefficient treatments for skin wound healing have always attracted the community’s attention because they cause a tremendous burden on patients, families, and society (Singer and Clark, 1999; Brown et al., 2008). Skin wound healing is a multi-process cascade that involves inflammation, hemagglutination, cell proliferation, and extracellular matrix (ECM) remodeling (Gurtner et al., 2008; Rodrigues et al., 2019). Delayed wound healing may occur due to a disorder in one of the four stages described earlier. During different stages, rapid closure of the wound site through migration and proliferation of epithelial cells is essential to restore barrier function (Takeo et al., 2015). In relation to re-epithelialization, the restoration of the dermis occurs by the proliferation and migration of fibroblasts (Driskell et al., 2013).

Human umbilical mesenchymal stem cells (hUMSCs), which can differentiate into cells, migrate to injured tissues and secrete many factors, show special advantages in medical applications due to their multipotent ability (Ding et al., 2011; Biazar and Keshel, 2013; Nakamura et al., 2013; Huang et al., 2020). However, there are still many obstacles to stem cell-based therapy; for example, the allogenic source and the tumorigenicity of these cells hinder their clinical application (Han and Sidhu, 2011; Kim et al., 2017). Exosomes (Exos) are extracellular vesicles with a size of 30–150 nm. Exosomes contain different proteins, lipids, nucleic acids, and various molecules (Kalluri and LeBleu, 2020). Evidence suggests that exosomes, as a cell-free therapy, can stimulate the local healing process *via* paracrine secretion (Tonnard et al., 2013; Bernardini et al., 2015). Exosomes isolated from hUMSCs have previously been shown to be capable of promoting skin wound healing (Chen et al., 2018; Tao et al., 2018; Henriques-Antunes et al., 2019). However, exosome isolation is still difficult and costly (Gurunathan et al., 2019). Recently, studies revealed that high-yield generation of exosome mimetics (EMs) can be substituted for exosomes for use in clinical practice, but this approach has not been applied for skin wound healing and tissue regeneration (Nasiri Kenari et al., 2020). Moreover, whether MSC-prepared exosome mimetics could promote wound healing, and the underlying mechanism in the treatment of skin injuries remain unclear.

**FIGURE 1:**
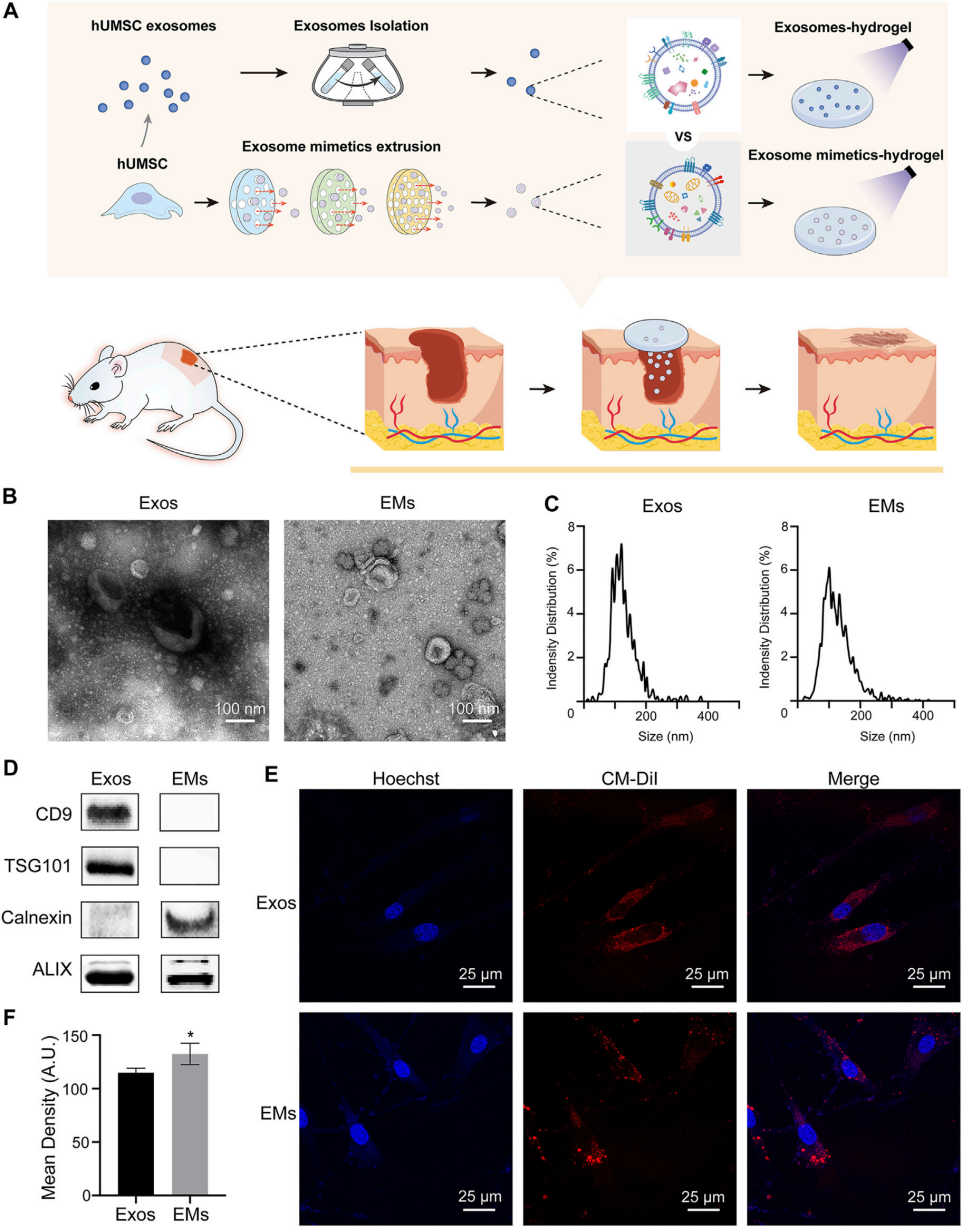
**(A)** Schematic overview of the development of hUMSC-Exos and hUMSC-EM hydrogels for skin wound healing and tissue regeneration. **(B)** TEM images of hUMSC-derived exosomes and exosome mimetics (hUMSC-derived exosomes: Exos; hUMSC-derived exosome mimetics: EMs). **(C)** Particle size distribution of hUMSC-derived exosomes and exosome mimetics measured by using nanoparticle tracking analysis (NTA). **(D)** Characteristic markers of hUMSC-derived exosomes and exosome mimetics. Membrane surface marker proteins (CD9), exosome biogenesis marker proteins (TSG101), endoplasmic reticulum related protein (calnexin), and endoplasmic marker proteins (ALIX) were analyzed by Western blotting. **(E)** Internalization of exosomes and EMs by fibroblasts. Exosomes and EMs are labeled in red, and fibroblast nuclei are labeled in blue. **(F)** The quantification of internalization. (*n* = 3, *p < 0.05)

As a biomaterial, hydrogels have already been widely used for various biomedical applications, such as drug delivery, tissue adhesion, and tissue regeneration (Pacelli et al., 2015). Gelatine methacryloyl (GelMA) hydrogels, with photo-crosslinking properties, resemble the properties of ECM based on their appropriate biological properties and tunable physical characteristics (Klotz et al., 2016). Drugs loaded on GelMA can be released slowly, improving the local drug administration concentration (Wang et al., 2019; Zhao et al., 2020). In this study, we investigated whether exosome mimetics could promote skin wound healing and suggested the underlying mechanism by defining proteomic differences (Figure 1A).

## Materials and Methods

### Cell Culture and Synthesis of Human Umbilical Mesenchymal Stem Cell-Derived Exosomes and Exosome Mimetics

All the cells were incubated at 37°C with 5% CO_2_, and hUMSCs were grown in a mesenchymal stem cell basal medium (Cell Farm, Shanghai, China) with 10% exosome-free fetal bovine serum (FBS) (Gibco). FBS was managed by ultracentrifugation at × 120,000 g for 120 min to remove the exosomes from FBS. All of the supernatant was collected from hUMSCs from passages 4 to 8. After collecting the supernatant of hUMSCs, the exosomes were ultracentrifuged at ×300 g for 10 min, ×2000 g for 10 min, and ×15,000 g for 40 min to remove cells and cell debris. Next, the supernatant was ultra-centrifuged at ×1,00,000 g for 70 min twice in Ultra-Clear centrifuge tubes (Beckman Coulter, United States) for exosome purification. Finally, the precipitate was resuspended in PBS and stored at −80°C for identification and the following experiments.

After hUMSCs reached a confluence of approximately 70%–80%, the cells were resuspended in a PBS solution of 1 × 10^6^ cells per 1 ml. The EMs were collected using serial extrusive approaches through polycarbonate membranes (Whatman, United States) of 10 nm, 5 nm, 1 nm, 800 μm, 400 μm, and 200 μm using a mini-extruder (Morgec, Shanghai, China). Exosome mimetics were centrifuged at × 100,000 g for 70 min twice and purified by a 100 kDa centrifugal filter (Millipore, CA, United States) at ×4,000 g for 20 min. The final EM sample was stored at −80°C.

### Identification of Human Umbilical Mesenchymal Stem Cell-Derived Exosomes and Exosome Mimetics

Transmission electron microscopy (TEM), ZetaView, and Western blotting analysis were applied to identify the characteristics of the isolated hUMSC-Exos. The morphology and microstructure of exosomes were observed using TEM (Jeol 1230, Japan). The size distribution and concentration of exosomes were measured at VivaCell Biosciences with ZetaView PMX 110 (Particle Metrix, Meerbusch, Germany) and the corresponding software ZetaView 8.04.02. Exosomal characteristic surface markers, including ALIX, CD9, TSG101, and calnexin, were analyzed by Western blotting analysis.

### Western Blotting Analysis

Exosome and EM solutions were used to isolate total cellular protein, and a BCA kit (Beyotime Biotechnology, Shanghai, China) was used for the quantification of protein levels. Proteins were separated by 10% SDS-PAGE, transferred to PVDF membranes, and finally probed with the appropriate primary antibodies specific for ALIX (#ab275377, Abcam, Cambridge, United Kingdom, 1:1000), CD9 (#ab236630, Abcam, Cambridge, United Kingdom, 1:1000), TSG101 (#ab125011, Abcam, Cambridge, United Kingdom, 1:1000), and calnexin (#133615, Abcam, Cambridge, United Kingdom, 1:1000). The hUMSC and EM solutions were used to isolate total cellular protein, and a BCA kit was used for the quantification of protein levels. Proteins were separated by 10% SDS-PAGE, transferred to PVDF membranes, and finally probed with the appropriate primary antibodies specific for cardiotin (sc-53002, Biocompare, 1:1000) and β-actin (#3700, CST, 1:1000). Then, we used secondary antibodies to probe the blots, and the proteins were detected with a Tanon 5200 scanner. The AllDoc_x software was used to quantify the protein band density.

### Exosome and Exosome Mimetics Uptake by Human Dermal Fibroblasts-a

To examine the internalization of hUMSC-derived exosomes by hDF-a, exosomes were labeled with a red fluorescent dye, CM-DiI (Sigma, United States). Then, labeled exosomes were incubated with hDF-a for 24 h. After that, the cells were fixed in 4% paraformaldehyde for 15 min and washed with PBS. The nuclei were stained with 1 μg/ml Hoechst (Invitrogen, United States) for 10 min before observation by using TEM.

### Preparation of GelMA Loaded With Human Umbilical Mesenchymal Stem Cell-Exosomes and Human Umbilical Mesenchymal Stem Cell-Exosome Mimetics

GelMA hydrogels (30% in LAP solution, EFL, Suzhou, China) were prepared according to the manufacturer’s instructions. Briefly, 3 g of gelatin was dissolved in 10 ml of LAP solution in a 55°C water bath for 30 min. Next, another 200 μg of exosomes or EMs were added to the mixed solution at room temperature. The mixed solution was irradiated under UV light for 1 min for crosslinking. To detect the distribution of exosomes in GelMA, exosomes and EMs were labeled with CM-DiI and then observed by TEM.

### Cell Counting Kit 8 Assay

CCK-8 assays were used to detect the effects of hUMSC-derived Exos and EMs on the proliferation of hDF-a cells. Briefly, hDF-a cells were seeded into 96-well plates at a density of 5 × 10^3^ cells per well and divided into seven groups when the cells were incubated until an exponential growth period. A complete high-glucose DMEM was used as the control (CN) group. The concentrations of exosomes and EMs were as follows: exosome 5 μg/ml (E5), exosome 20 μg/ml (E20), exosome 50 μg/ml (E50), EMs 5 μg/ml (EM5), EMs 20 μg/ml (EM20), and EMs 50 μg/ml (EM50). After being treated with different concentrations of exosomes and EMs for 1 day, 90 μl of culture medium mixed with 10 μl of CCK-8 reagent (Dojindo, Japan) was added into each well and incubated with 5% CO2 at 37°C for 2 h in a humidified incubator. Then, the optical density (OD) value at 450 nm was measured by the microplate reader (Tecan, Thermo Scientific, United States).

### EdU Proliferation Assay

An EdU kit (Riobo, Guangzhou, China) was used to detect the effects of hUMSC-derived Exos and EMs on the proliferation of hDF-a cells. The hDF-a cells were seeded at 1 × 10^5^ cells per well in 24-well plates and were cultured until the exponential growth period. Then, the cells were divided into seven groups as follows: the control (CN) group, exosomes 5 μg/ml (E5), exosomes 20 μg/ml (E20), exosomes 50 μg/ml (E50), EMs 5 μg/ml (EM5), EMs 20 μg/ml (EM20), and EMs 50 μg/ml (EM50). The control group of cells was cultured in high-glucose DMEM. Moreover, the exosomes and EM groups were prepared with a concentration gradient. After the cells were cultured for 24 h, the EdU experiment was performed following the manufacturer’s instructions. Cell proliferation was determined by counting the number of EdU-positive cells and total cells from randomly selected image fields. Then, the ratio of EdU-positive cells to total cells was calculated. Images were taken with a Zeiss fluorescence microscope (Axio Observer.D1, Shanghai, China).

### Scratch Wound Assay

Briefly, 1 × 10^5^ cells per well were seeded into 6-well plates and incubated for 24 h. Then, the confluent layer of cells was scratched using a sterile 20–200 μL pipette tip. After washing the cells with PBS, 0, 20, or 50 μg/ml exosomes or EMs were added. Images were recorded at 0, 6, and 12 h after the monolayers were scratched. Scratched areas were measured by ImageJ software.

### *In Vivo* Cutaneous Wound Healing Model of Mice

C57 mice (22 ± 2 g) at 8 weeks old were purchased from Shanghai Jihui Laboratory Animal Technology Co., Ltd. (Shanghai, China). Animals were housed in an SPF-class laboratory and allowed access to water and food. All animal care and experimental procedures were supported by the Committee on Ethics of Biomedicine, Naval Medical University (Approval No: 20180309020). Before the surgery, the mice were anesthetized by an intraperitoneal injection of 2% pentobarbital (50 mg/kg). After shaving the dorsal hair and disinfecting the skin, two 10 mm × 10 mm bilateral symmetry round full-thickness incisions were made by a 10-mm punch biopsy on the upper back. The mice were randomly divided into five groups of six animals as follows: 1) Control group: the wounds were merely covered by gauze and Tegaderm™ film (3M, United States) without any treatment. 2) Exos group: the 20 μg/ml hUSMC-Exos were injected subcutaneously around the wound area. 3) Exosome mimetics group: the 20 μg/ml hUSMC-EMs were injected subcutaneously around the wound area. 4) GelMA-Exos group: the wounds were covered by GelMA loaded with 20 μg/ml hUSMC-Exos. 5) GelMA-EMs group: the wounds were covered by GelMA loaded with 20 μg/ml hUSMC-EMs. The wounds were photographed at 0, 4, 7, and 14 days after surgery. The wound size was measured by ImageJ software (NIH, United States). At 4, 7, and 14 days after surgery, two mice in each group were sacrificed for further analysis.

### Histology Analysis

The wounded skin in each group was removed at days 4, 7, and 14 postsurgery, fixed in 4% paraformaldehyde for 48 h, gradually dehydrated with a series of graded ethanol solutions, embedded in paraffin, and sectioned into 5 μm-thick sections. Then, the sections were stained with hematoxylin and eosin (H&E) and Masson’s trichrome. H&E staining was used to evaluate the extent of wound healing, while Masson’s trichrome staining was performed to determine the maturity of collagen. Cell proliferation was tested by immunohistochemical staining of Ki67 (AF0198, Affinity Biosciences, United States). The sections were rehydrated and heated in a microwave in citrate buffer (0.01 M; pH 6.0) for 15 min to retrieve the antigen. Then, they were blocked in 1% BSA for 30 min at room temperature, incubated with the primary antibody anti-ki67 overnight at 4°C, and finally, they were incubated with the respective secondary antibodies at room temperature for 1 h in the dark. Images were acquired with a fluorescence microscope.

### Proteomic Profiling

Liquid chromatography–tandem mass spectrometry (LC-MS/MS)-based proteomic technologies were used to ionize and separate eluted peptides by mass spectrometry according to their unique mass-to-charge ratio (m/z). The data were analyzed in Proteome Discoverer (v2.4.1.15) software (Thermo Fisher) for the main search. The differential expression threshold was set to a 1.5 fold change. After the t-test analysis, significantly differentially enriched proteins were identified through volcano plot filtering between the two experimental groups. Finally, hierarchical clustering was performed to show distinguishable enriched protein profiles among the samples using the GO and KEGG databases (http://eggnog5.embl.de).

### Statistical Analysis

The data are presented as the mean ± standard deviation (SD). Two groups were compared by Student’s t-test using GraphPad Prism 8.0 software. A two-sided t-test with *p* < 0.05 was defined as statistically significant.

## Results

### Characterization of Human Umbilical Mesenchymal Stem Cell-Derived Exosomes and Exosome Mimetics

hUMSC exosomes were collected using ultracentrifugation as previously described (Livshits et al., 2015; Li et al., 2017). Then, hUMSCs were extruded to form EMs, which were collected by using ultracentrifugation (Huang et al., 2020; Nasiri Kenari et al., 2020).

hUMSC-derived exosomes and EMs were characterized by TEM, NTA, and Western blotting analysis. TEM showed the morphologies of hUMSC-derived exosomes and EMs as shown in Figure 1B. We can see that EMs, which are the same as exosomes, are round cup-shaped vesicles with lipid membranes (Kalluri and LeBleu, 2020). The NTA results (Figure 1C) of hUMSC-derived exosomes and EMs demonstrated an approximate Gaussian distribution of size with a peak diameter of approximately 100 nm, which is consistent with the TEM results. From Western blotting analysis, vesicle membrane markers (CD9), multivesicular body proteins (TSG101), and cell generation-related protein (ALIX) (Kalluri and LeBleu, 2020) are used as identification proteins for exosomes. In addition, EMs had endoplasmic reticulum-related protein (calnexin) which exosomes did not have (Figure 1D) (Chen et al., 2020).

Previous work verified that exosomes and EMs have similar characteristics and extensive biological effects (Lu and Huang, 2020). After that, the uptake efficacy of exosomes and EMs by recipient cells, such as dermal fibroblasts, was also evaluated. We labeled the hUMSC membrane with CM-DiI in red and then introduced it into polycarbonate filters with pores to produce EMs. At the same time, the exosomes were also labeled with CM-DiI. Exosomes and EMs were incubated with the human dermis-immortalized fibroblast cell line hDF-a for 24 h separately (Zhang et al., 2016). After Hoechst staining of the nuclei in blue, the TEM merging graphics showed that these two kinds of nanoparticles could be internalized into the cells (Figure 1E). The obtained results indicate that hDF-a has effective internalization capabilities for both hUMSC-Exos and EMs.

### Human Umbilical Mesenchymal Stem Cell-Derived Exosome Mimetics Can Promote Proliferation and Migration of Human Dermal Fibroblasts-a *In Vitro*

**FIGURE 2:**
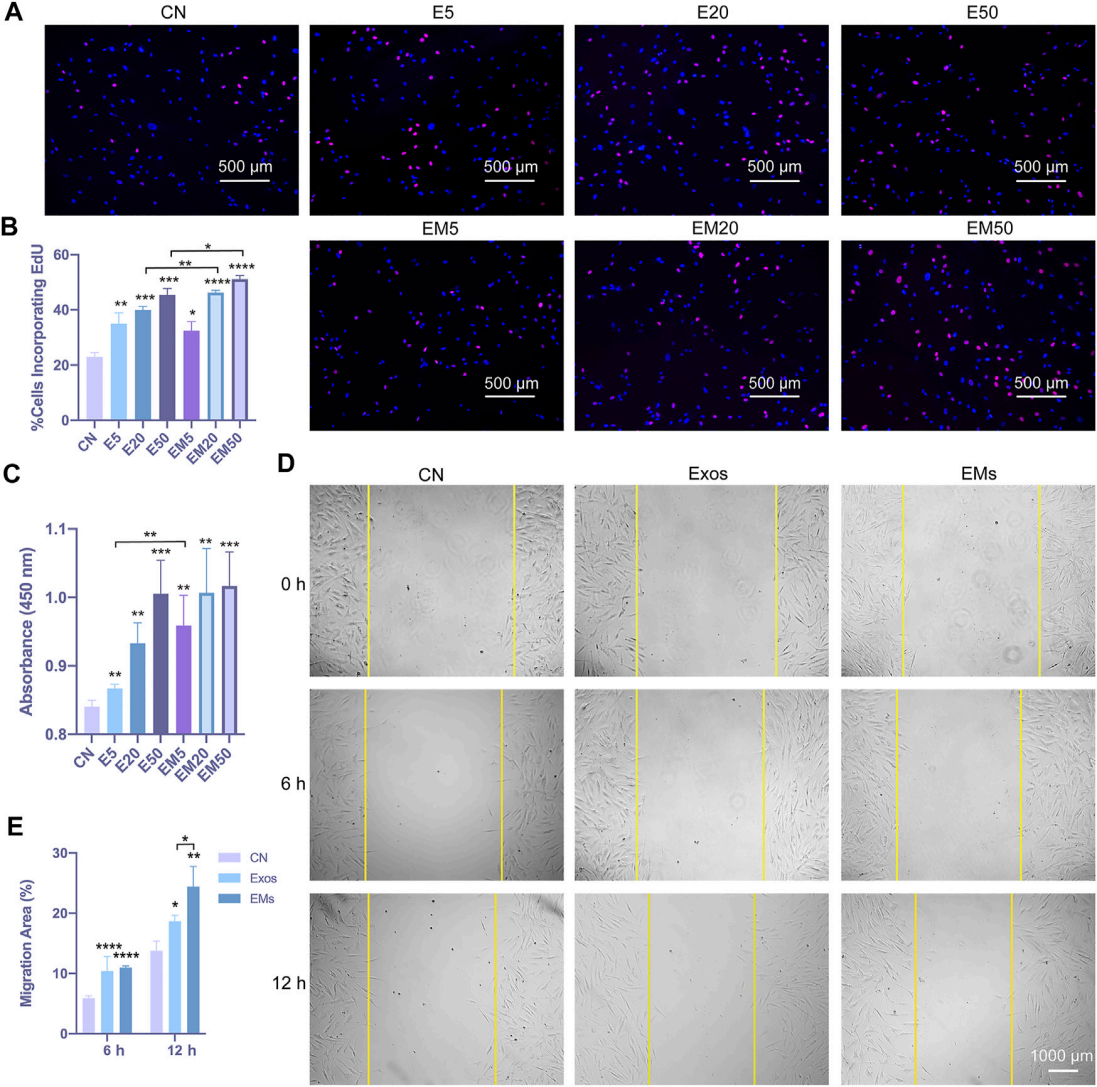
The effect of hUMSC-derived exosomes and EMs on the proliferation and migration of hDF-a cells *in vitro* (CN = control, E5 = exosome 5 μg/ml, E20 = exosome 20 μg/ml, E50 = exosome 50 μg/ml, EM5 = EMs 5 μg/ml, EM20 = EMs 20 μg/ml, EM50 = EMs 50 μg/ml). **(A)** hUMSC-derived exosomes and EMs promoted proliferation of hDF-a by EdU incorporation assays. Cell nuclei were stained blue. After the images are merged, proliferating cells were counterstained purple with an EdU probe. **(B)** Quantitative analysis of the EdU incorporation assays (*n* = 4, *p < 0.05, **p < 0.01, ***p < 0.001, ****p < 0.0001). **(C)** hUMSC-derived exosomes and EMs promoted the proliferation of hDF-a cells as shown in CCK-8 assays. (*n* = 4, *p < 0.05, **p < 0.01) **(D)** A scratch assay verified the migration of hDF-a cells *in vitro* by hUMSC-derived exosomes and EMs. **(E)** Quantitative analysis of scratch assays (*n* = 4, *p < 0.05, **p < 0.01, ****p < 0.0001).

Next, the regulation of hDF-a behavior by exosomes and EMs was evaluated. The EdU incorporation assay and CCK-8 assays were used to evaluate hDF-a proliferation ability, and the scratch test was used to evaluate hDF-a migration ability (Liu et al., 2018). With increasing concentrations of exosomes and EMs, the number of EdU-probe-labeled proliferating cells per field was gradually increased in a dose-dependent manner compared to the exosome group and control group (Figure 2A). Moreover, the EMs group showed higher proliferative capability than the exosome group at a certain concentration (Figure 2B). After 4 h of incubation, as shown by the OD values at 450 nm, the results of the CCK-8 assays (Figure 2C) also showed the same tendency as the EdU incorporation assays. This result indicated that the proliferation-promoting effects of exosomes and EMs on hDF-a were equal at the same concentration.

We also applied the scratch assay to verify the migration of hDF-a cells *in vitro* after hUMSC-derived exosome or EM treatments, and the result indicated that exosomes and EMs could promote the migration of hDF-a (Figure 2D). The quantitative analysis by ImageJ suggested that the percentage of migration area (%) of EMs was slightly smaller than that of exosomes (Figure 2E). In this part, the promoting effects of exosomes and EMs on the proliferation and migration of fibroblasts were tested, and EMs showed equal effects compared with exosomes.

### Human Umbilical Mesenchymal Stem Cell-Derived Exosome Mimetics Loaded on GelMA Accelerate Wound Healing *In Vivo*

**FIGURE 3:**
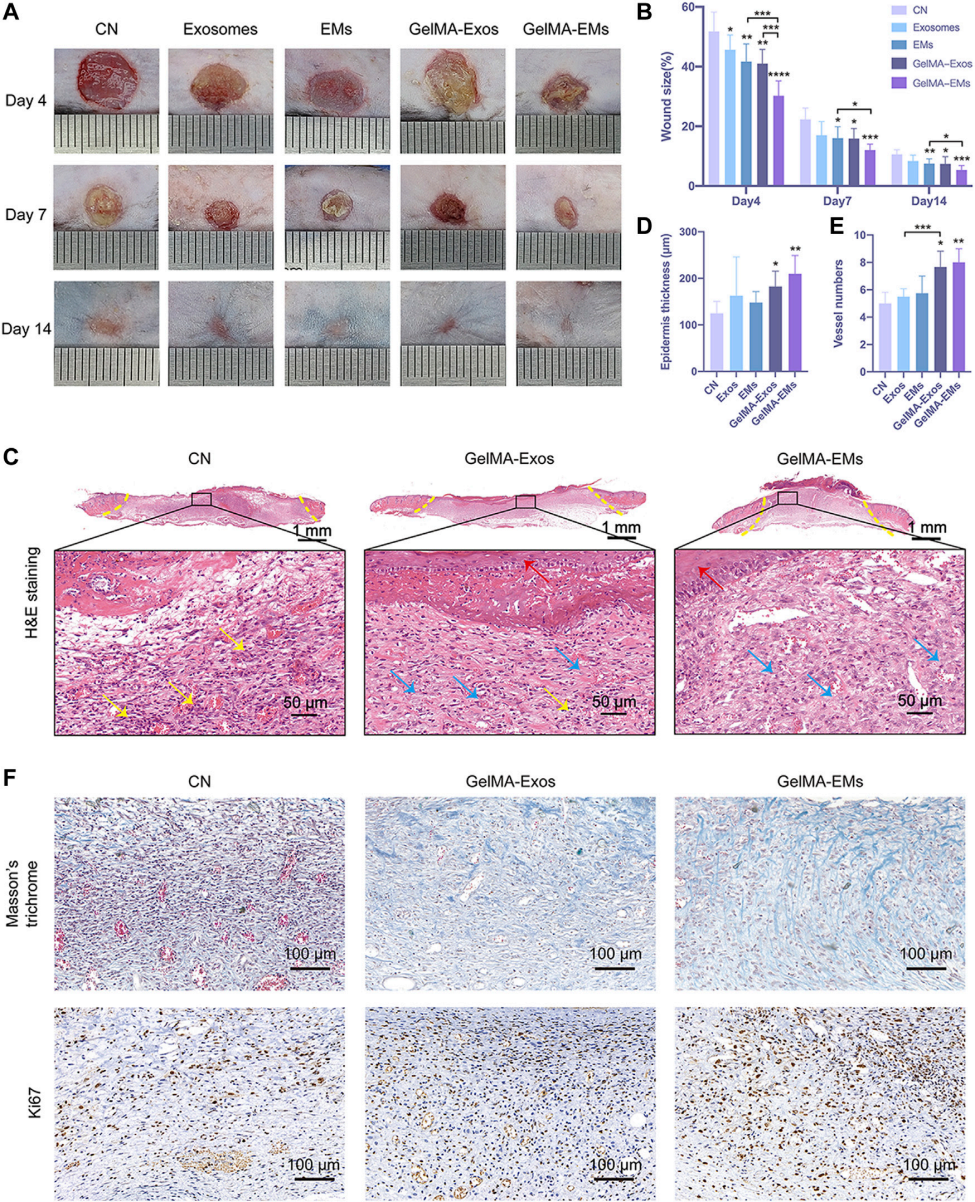
The effect of hUMSC-derived exosome mimetics on skin wound healing *in vivo*. Representative images **(A)** and quantification **(B)** of full-thickness cutaneous wound sizes showed that the hUMSC-derived exosome mimetics loaded on GelMA could accelerate the wound healing process. The data are shown as the mean ± SD. The significant difference between the untreated and hydrogel-treated groups was analyzed by the t-test (*n* = 6, *p < 0.05, **p < 0.01, ***p < 0.001, ****p < 0.0001). Epidermis thickness **(C)** and vessel numbers **(D)** per random field were analyzed by the t-test. (*n* = 4, *p < 0.05, **p < 0.01) H&E staining **(E)** of the wound sites is shown. The yellow dotted lines indicate the boundary of the wound sites. The blue arrows indicate granulation tissues. The yellow arrows indicate inflammatory neutrophils. The red arrows label the newly formed epidermis. **(F)** Masson’s trichrome staining and ki67 immunohistochemistry staining of wound sites.

The effects of exosomes and EMs on wound healing were subsequently evaluated. hUMSC-derived exosomes and EMs were labeled with CM-DiI and then loaded on GelMA. Exosomes and EMs were both evenly incorporated into GelMA (Supplementary Figure S1). In addition, we investigated whether the hUMSC-derived EM-loaded GelMA hydrogel could improve the closure and tissue repair of full-thickness skin wounds (Supplementary Figure S2). Figure 3A shows optical images of the wound healing on days 4, 7, and 14 after surgery. While the wound sizes in all five groups decreased with time, especially on day 4 after injury, the wound sizes in the GelMA-EMs group were clearly smaller than those in the GelMA-Exos group, and the wounds had almost closed by 14 days. In addition, the EMs group had a repair effect similar to that of the exosomes group. The exosomes group showed a better repair effect than the control group as previously described. Quantification of the wound sizes confirmed the aforementioned results (Figure 3B).

Hematoxylin and eosin (H&E) staining showed the histologic changes of the wounds at day 7 (Figure 3C). The yellow dotted lines indicate the boundary of the wound site. Compared to the control group, we can see that the GelMA-EMs group showed a reduced inflammatory response, and the granulation tissues were restored much more quickly. The area of granulation tissues in random sights differs remarkably. The newly formed epidermis was nearly intact and stretched under hydrogel residues in the GelMA-Exos and GelMA-EMs groups. The thickness (Figure 3D) of the neo-epithelium of GelMA-EMs was significantly increased. The vessel numbers in random 0.04 mm^2^ fields (Figure 3E) indicate that the wound bed of the experimental groups has higher angiogenesis activity than that of the control group. It is known that the fibroblasts showed higher proliferation activity 7 days after injury. Afterward, macroscopic images of Masson’s trichrome staining were applied to evaluate the deposition and maturation of collagen on day 7 (Figure 3F). Ki67 immunohistochemistry was used to investigate cell proliferation (Yang et al., 2020). Compared to the other four groups, the experimental groups showed stronger positive staining of Ki67 (Supplementary Figure S3), suggesting that both exosomes and EMs could facilitate cell proliferation during the healing period (Supplementary Figure S3), thereby accelerating granulation tissue formation and enhancing collagen deposition. The *in vivo* experiments showed that GelMA-loaded EMs could promote skin wound healing, and these results provide promising prospects for clinical application.

### Proteomic Profiling Reveals a Distinct Protein Landscape Between Exosome Mimetics and Exosomes

**FIGURE 4:**
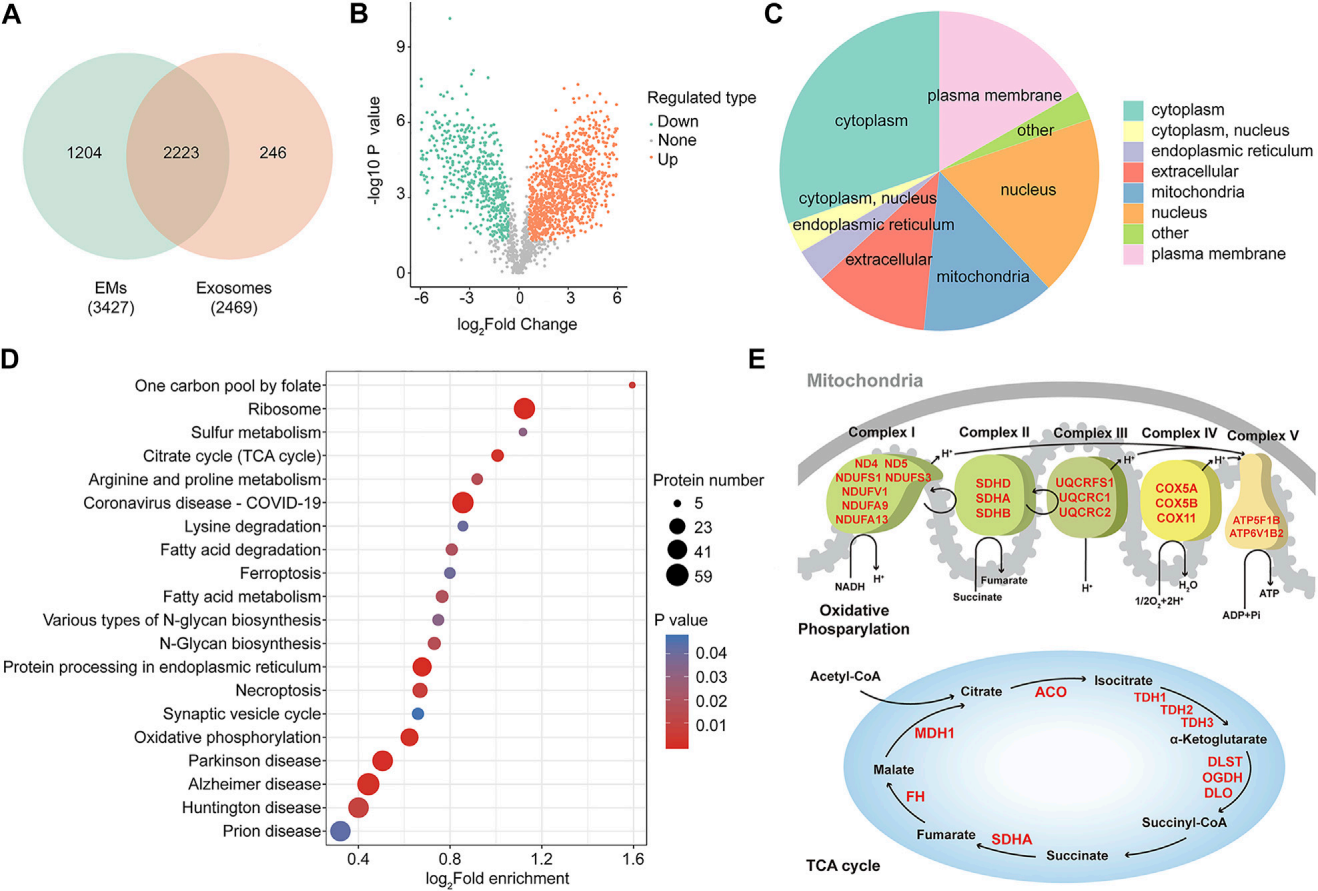
Proteomic profiling reveals a distinct protein landscape between EMs and exosomes. **(A)** Venn diagram showing the number of identified proteins of hUMSC-derived exosomes and EMs (*n* = 3). **(B)** Volcano plots are useful to visualize differentially enriched protein patterns among hUMSC-derived EMs and exosomes (the quantitative difference in enriched protein between EMs to exosomes using *p*-value > 0.05, FC ≥ 1.5). **(C)** Subcellular analysis of differentially enriched protein annotations between EMs and exosomes. **(D)** KEGG enrichment analysis of differentially enriched proteins annotations between EMs and exosomes. **(E)** Upregulated protein enriched in mitochondrial oxidative phosphorylation and the TCA cycle.

The aforementioned data show that compared with hUMSC-derived exosomes, hUMSC-derived EMs have a similar efficiency in wound healing and even better efficiency under some circumstances. To determine the molecular difference between exosomes and hUMSC-derived EMs, we carried out label-free mass spectrometry-based proteomics. Proteomic profiling identified 2,469 proteins in exosomes and 3,427 proteins in EMs from a total of 3,673 proteins. A number of proteins (2,223) were commonly found in both samples (Figure 4A). The differentially enriched protein between samples was examined by the t-test and *p* values, indicating that 1,666 proteins were significantly differentially enriched (FC > 1.5, *p* < 0.05). A volcano plot was generated to visualize the differential enrichment between the two groups, as shown in Figure 4B. Next, the upregulated proteins in hUMSC-derived EMs were further analyzed based on the Gene Ontology (GO) and the Kyoto Encyclopedia of Genes and Genomes (KEGG) databases (http://eggnog5.embl.de) (Huerta-Cepas et al., 2019). Subcellular analysis of upregulated proteins in hUMSC-derived EMs was performed (Figure 4C). Interestingly, in addition to the extracellular matrix, cytoskeleton, and cell membrane, many proteins were localized in the mitochondria. Furthermore, the results of enrichment analysis of the KEGG signaling pathway showed that EM proteins were enriched in oxidative phosphorylation- and tricarboxylic acid cycle (TCA cycle)-related pathways (Figure 4D). Subsequent analysis of the proteomic data revealed that EMs-upregulated proteins are mainly concentrated in the enzymes related to material metabolism in the TCA cycle and the respiratory chain complex (Figure 4E, red) (Fernie et al., 2004). Taken together, we showed that distinguishable protein profiles between EMs and exosomes are upregulated, especially in the mitochondria and oxidative phosphorylation. Protein profiling also revealed that EMs may promote wound healing by enhancing the oxidative phosphorylation of cells in the wound.

## Discussion

Delayed wound healing remains an intractable affair for patients, families, and society. Recent studies of hUMSC-derived exosomes showed that, in addition to their cell communication function, they also play an important role in transferring therapeutic factors. The benefit of exosomes is that they have quantities of bioactive substances and lower immunological responses than the cells (Chen et al., 2018; Tao et al., 2018; Henriques-Antunes et al., 2019). The application of exosome-based cell-free therapy in wound healing and tissue regeneration attracts our attention. However, exosome-based therapy is still limited by their heterogeneity, therapeutic loading, large-scale manufacturing restrictions, and low yield for extraction and purification (Gurunathan et al., 2019). Recently, engineering nanoparticles are said to be obtained by polycarbonate filters with pores, microfluidic devices, supramolecular chemistry, and hybridization (Nasiri Kenari et al., 2020; Li et al., 2021). New studies have shown that EMs, as a kind of engineered nanoparticle, demonstrate reproducibility in the protocol, cost-effectiveness, higher yield production rates, and can simulate complex biological components (Ilahibaks et al., 2019; Li et al., 2021). Furthermore, Fan and his colleagues showed that hUMSC-derived EMs could promote bone regeneration (Fan et al., 2020). Therefore, we wondered whether the EMs could promote skin wound healing and tissue regeneration and whether there was a difference between extruded exosome mimetics and natural exosomes.

In this study, hUMSC-derived EMs were achieved by taking hUMSCs through polycarbonate filters with hUMSC-derived exosomes as their controls. As described in the results, we found that hUMSC-derived exosomes and EMs shared a similar appearance, indicating that EMs could potentially take advantage of nanoparticle properties such as biocompatibility, biological stability, and plasma protein interactions. Because EMs were eventually extruded by filters of 200 nm, compared to exosome particle sizes concentrated at approximately 100 nm, the particle sizes of EMs were concentrated between 100 and 200 nm. Moreover, besides the traditional cell membrane markers similar to exosomes, EMs also enriched some other proteins from their donor cells, which were shown in our Western blotting analysis. These proteins were also identified by mass spectrometry-based proteomics analysis. Biocompatible markers, such as CD55 and CD59, may help EMs escape from elimination and prolong the half-life of circulation.

GelMA hydrogels have been reported as promising biomaterials to deliver drugs/cells for wound healing and tissue regeneration because they have inherent advantages, such as absorbing wound fluid, preventing infection, and offering gaseous exchange (Pacelli et al., 2015). It could be easily achieved by conjugating lithium phenyl-2,4,6-trimethylbenzoylphosphinate (LAP) with GelMA by shielding it under UV rays (Yue et al., 2015). To increase the local drug distribution and allow exosomes and EMs to be continuously released, GelMA could be an ideal biomaterial. We successfully assembled exosomes and EMs on GelMA and they could be distributed evenly. Therefore, we proposed that bioactive hydrogels carrying hUMSC-derived EMs could be a highly efficient novel method for wound healing and tissue regeneration.

As shown in the results, EMs promoted fibroblast proliferation and migration *in vitro*. Surprisingly, EMs have even better biological activities than exosomes under certain conditions. At the same time, the results *in vivo* showed a similar result that the EMs could promote wound healing as effectively as exosomes, and the GelMA-EMs group presented the best wound healing efficiency even over the GelMA-Exos group. The skin wound injury treatment outcomes were especially diverse regarding wound area shrinkage, tissue regeneration, and epidermal stretching. The manufacturing of engineered exosome mimetics, an alternative candidate of exosomes to mimic exosomes by size, morphology, and function, has been proposed in this article. The administration of exosomes and EMs by GelMA has better efficiency than mere subcutaneous administration. The obtained results suggested that EMs combined with wound dressing scaffolds could provide a novel cell-free method for wound healing and tissue regeneration.

Although the benefits of hUMSC-derived EMs address the issues in wound healing, the proteome cargo of EMs has not been comprehensively characterized and may potentially have an effect on recipient tissues and cells (Nasiri Kenari et al., 2019). We investigated the proteome cargo of EMs and compared it to that of natural exosomes secreted from the same cell line. A subset of proteins is highly abundant in hUMSC-derived EMs compared to well-known exosomal cargo proteins. We further applied GO and KEGG pathway analyses. There was a noteworthy enrichment of the mitochondrial-related proteins in EMs compared to exosomes. Therefore, we evaluated the content of mitochondrial-related proteins in EMs, and the results showed that there were abundant mitochondrial-related proteins in EMs (Supplementary Figure S4) (Driesen et al., 2009). The aforementioned results indicate that the functional mitochondrial components in hUMSCs may be packaged into EMs. As it is well known, the mitochondrion is an energy reservoir for the functional ability and individual activity. Hayakawa and his colleagues reported that astrocytic mitochondria could provide entry to adjacent neurons and amplify cell survival signals (Hayakawa et al., 2016). Many researchers have reported that mitochondrial transfer from the bone marrow-derived stromal cells could protect against acute lung injury (Islam et al., 2012), and it is envisaged that EMs may take effect in the means of MSC mitochondrial transfer to regulate wound healing and tissue regeneration processes.

## Conclusion

In this study, the efficiency of EMs in repairing wounds was verified *in vivo* and *in vitro*. The obtained results showed that compared with hUMSC-derived natural exosomes, hUMSC-derived exosome mimetics could similarly promote wound healing by promoting the proliferation and migration of dermal fibroblasts. Further proteomics data revealed that EMs carry mitochondrial-related proteins derived from hUMSCs. Quantities of TCA cycle-related proteins and respiratory chain complexes are significantly enriched in EMs. The obtained results suggest that treatment with GelMA-carrying EMs for wound healing and tissue regeneration could have promising prospects for future clinical applications. EMs are promising candidates for natural cell-derived exosomes.

## Data Availability

The Proteomic raw data are available in iProX (www.iprox.org) under the accession number IPX0004319000.
