# Supplementary material for: Exosome Mimetics-Loaded Hydrogel Accelerates Wound Repair by Transferring Functional Mitochondrial Proteins
Source: Front Bioeng Biotechnol. 2022 May 20;10:866505. doi: 10.3389/fbioe.2022.866505 (PMC9163684; doi:10.3389/fbioe.2022.866505)

**Supplementary**


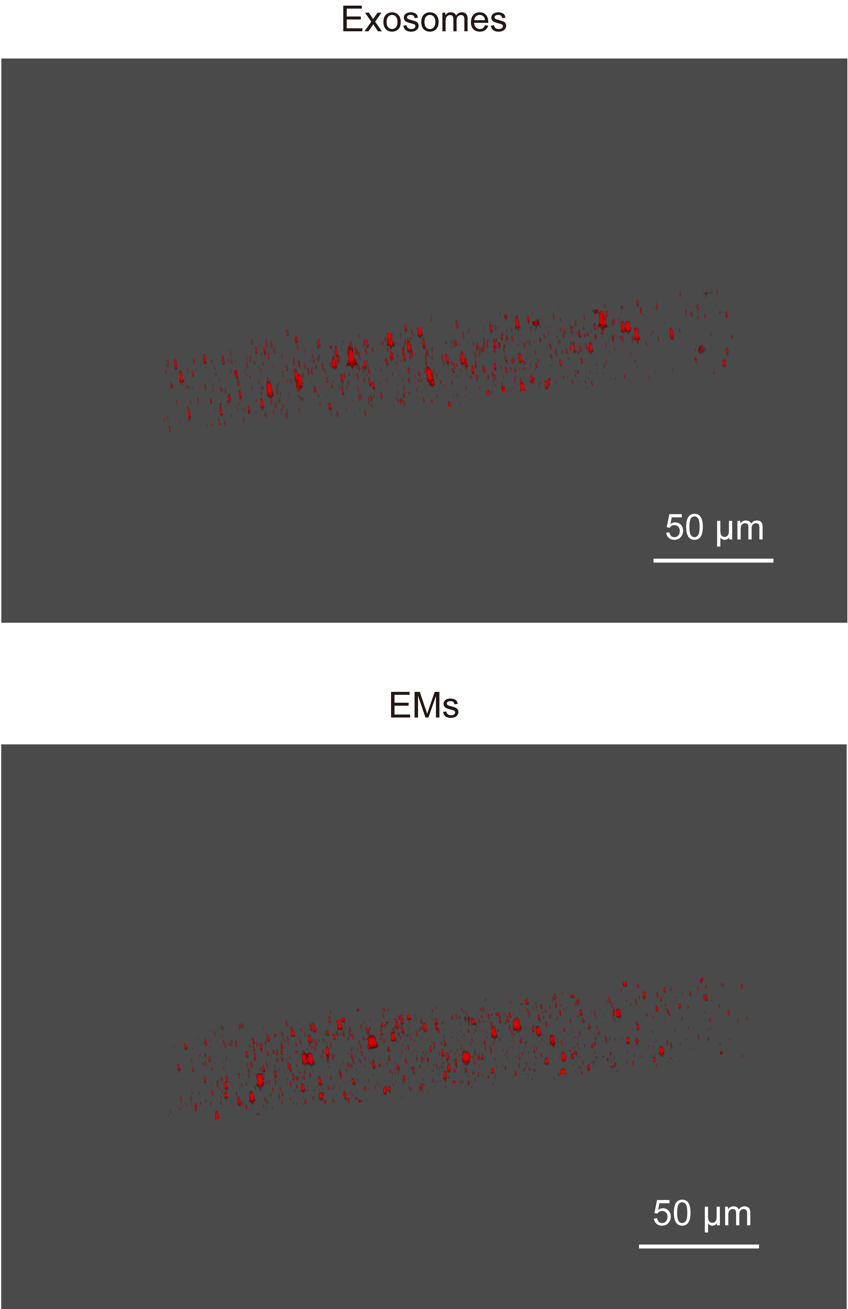


**Supplementary Figure 1**. TEM images showed that exosomes and EMs could both incorporate on GelMA evenly.


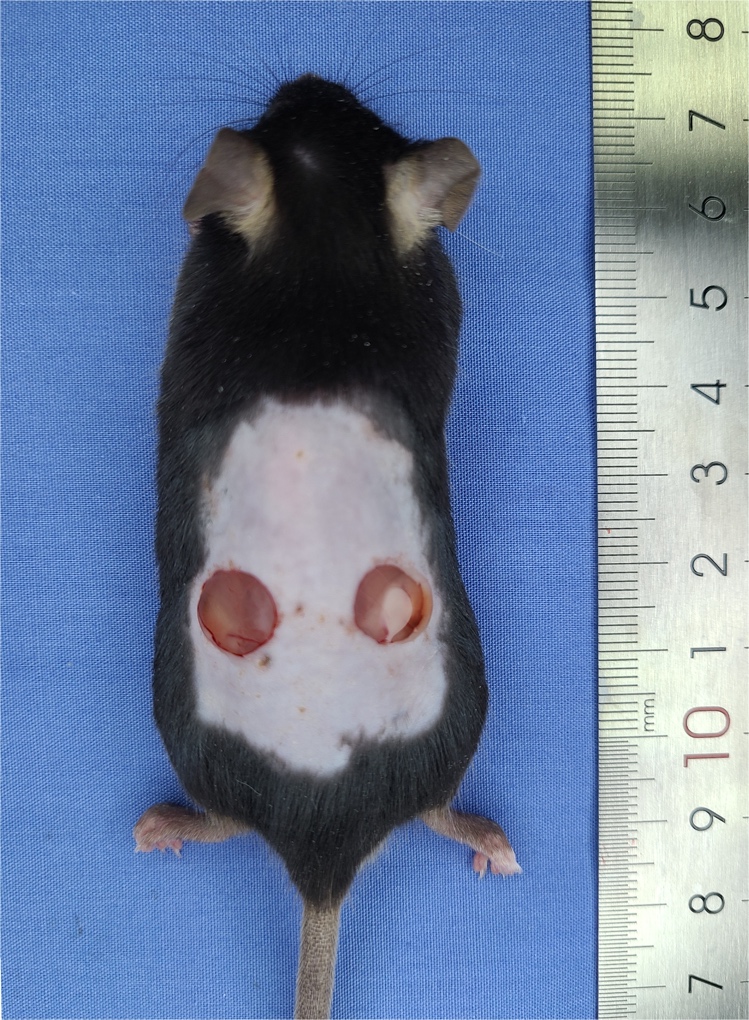


**Supplementary Figure 2**. The exosomes-hydrogel and exosome mimetics-hydrogel covered on the wound bed in vivo.


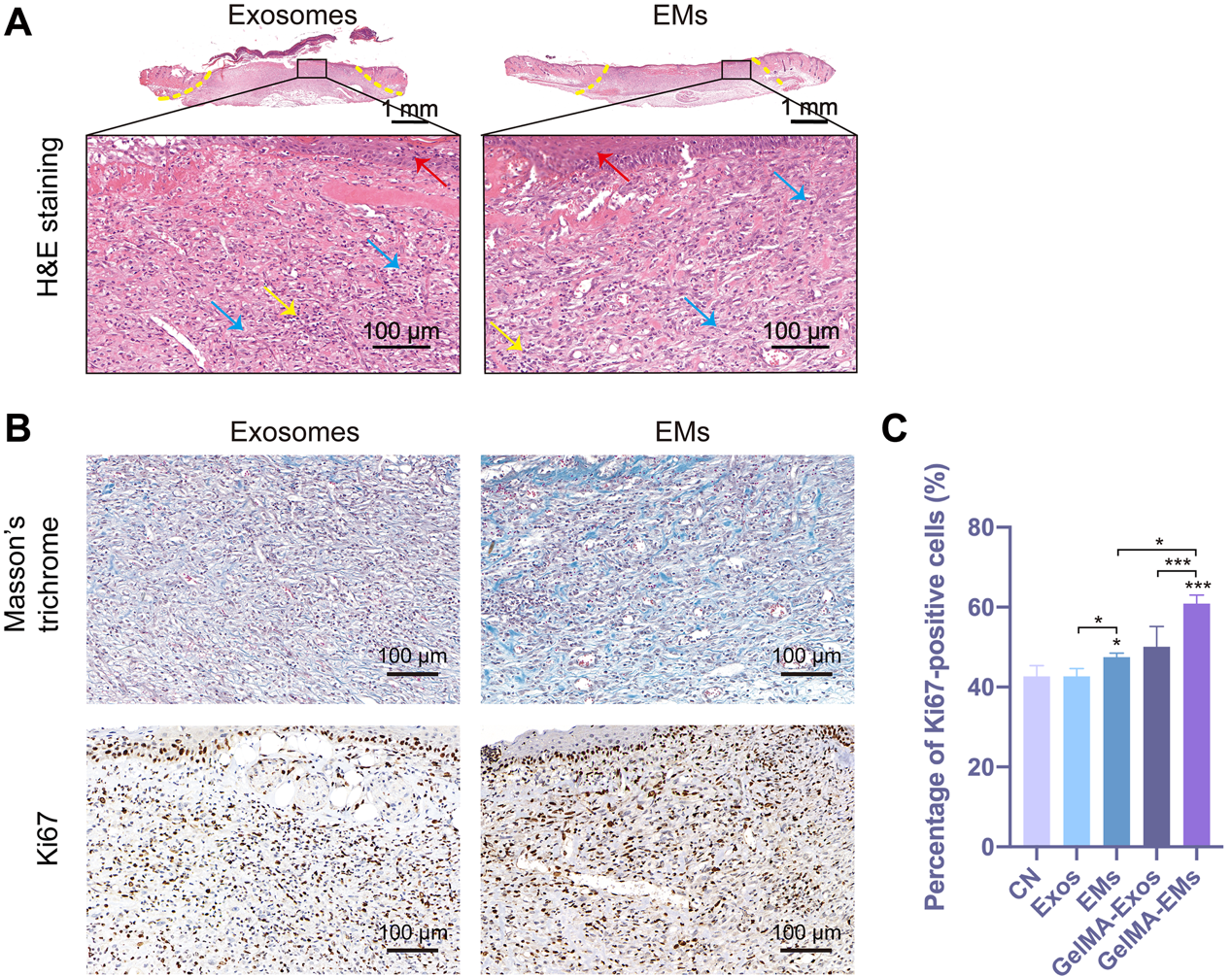


**Supplementary Figure 3**. H&E and immunohistology staining showed that EMs have equally efficiency with Exosomes on wound healing. (n=3, **p* <0.05, ****p* < 0.001, *****p* < 0.0001)


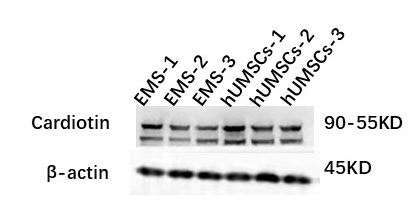


**Supplementary Figure 4**. Western blot results of hUMSCs and EMs.

**Graphic abstract**


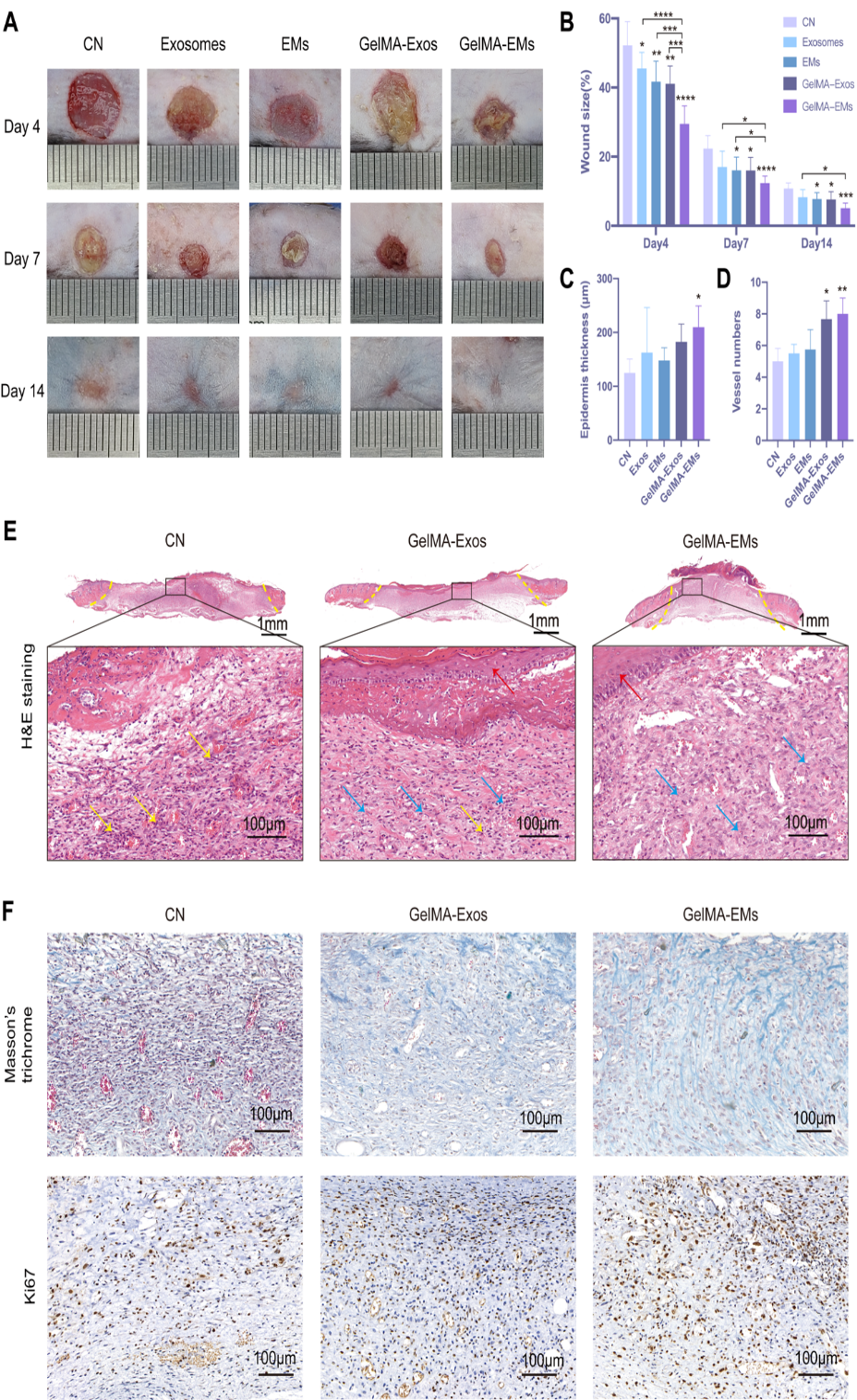

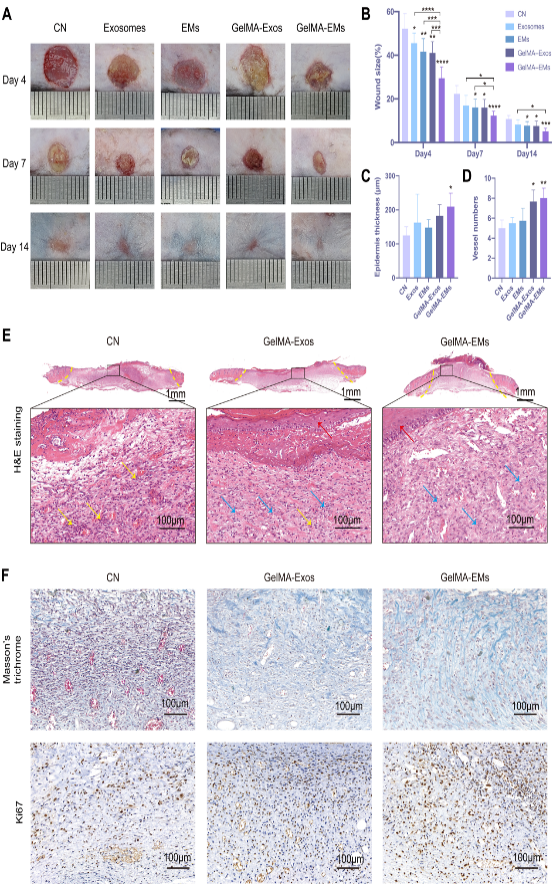

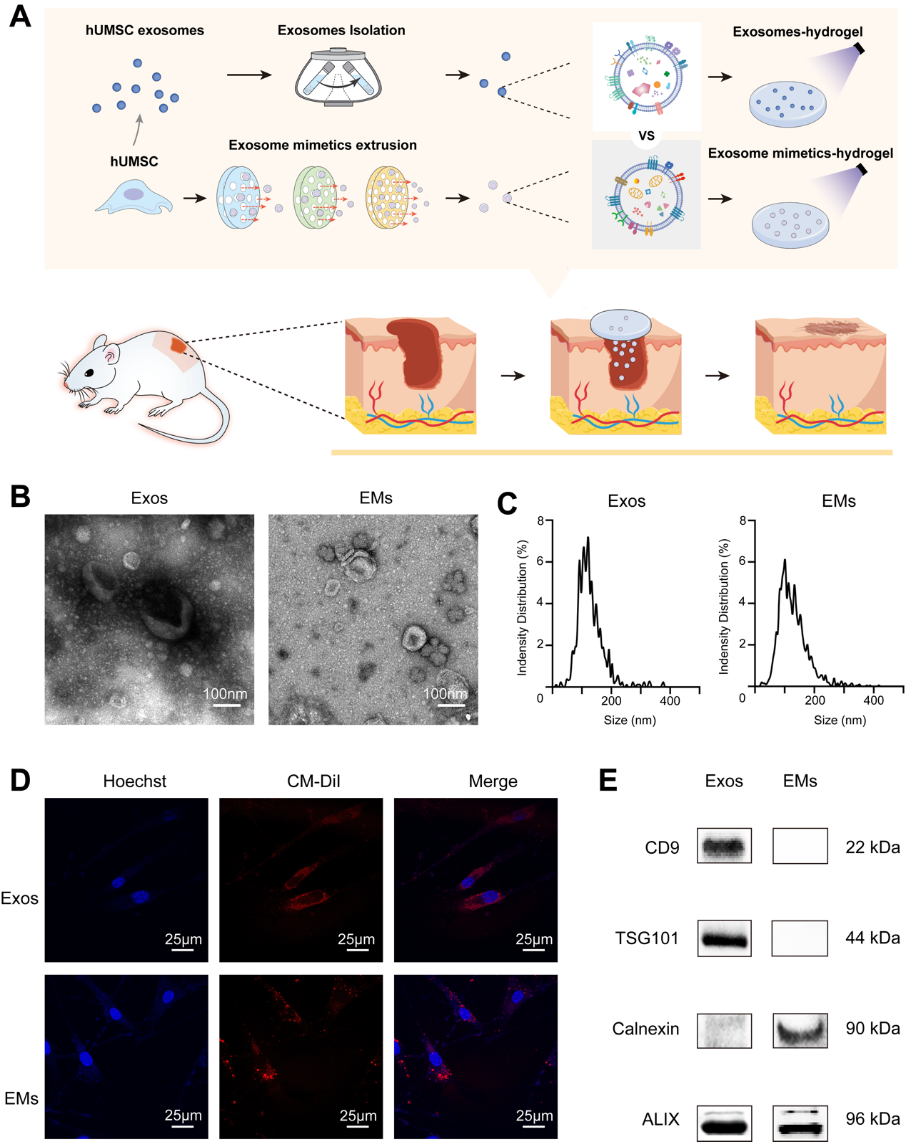

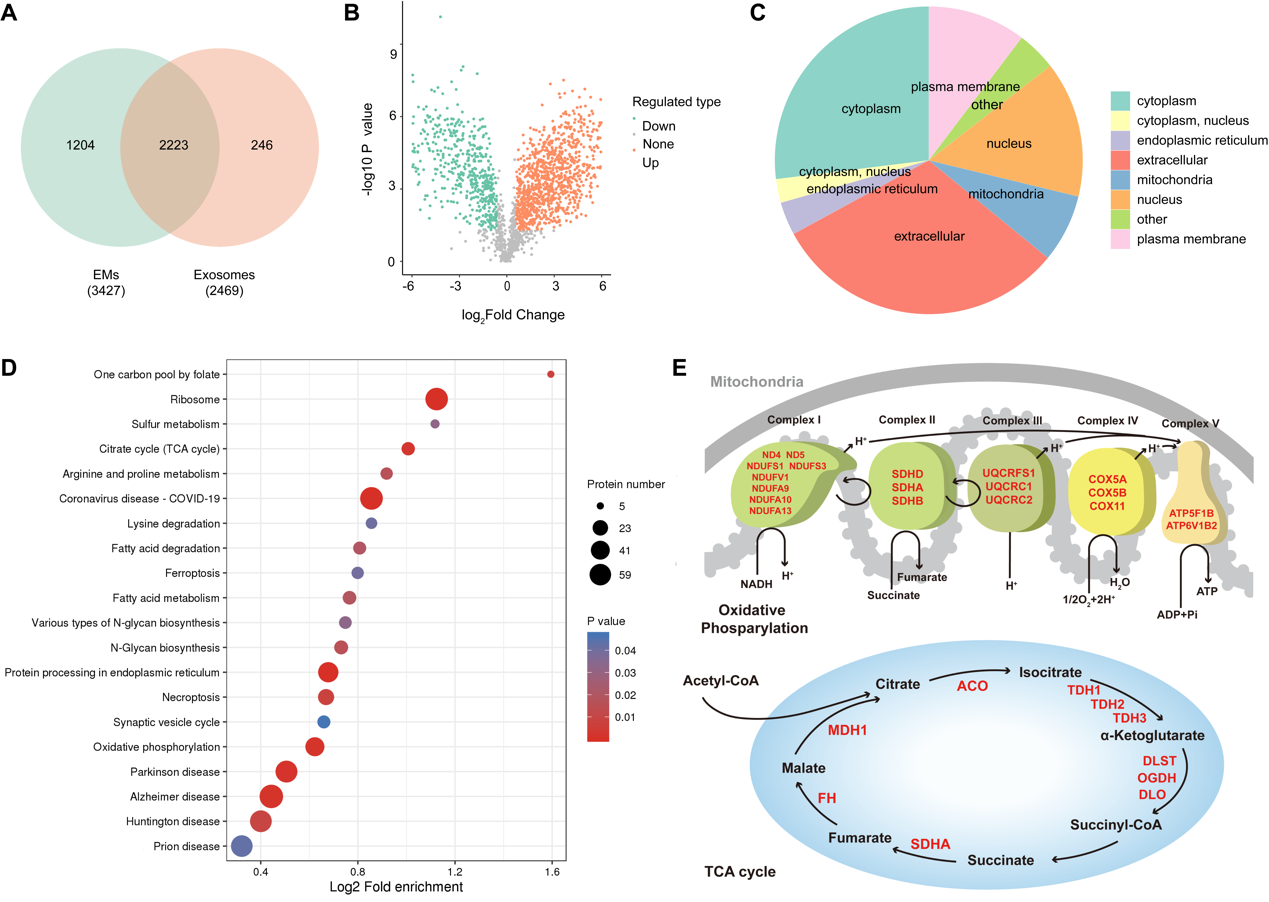

Supplement: Supplementary file 1 [file Table1.DOCX]
